# Composite activity type and stride-specific energy expenditure estimation model for thigh-worn accelerometry

**DOI:** 10.1186/s12966-024-01646-y

**Published:** 2024-09-10

**Authors:** Claas Lendt, Niklas Hansen, Ingo Froböse, Tom Stewart

**Affiliations:** 1 Institute of Movement Therapy and Movement-oriented Prevention and Rehabilitation, German Sport University Cologne, Cologne, Germany; 2 Human Potential Centre, School of Sport and Recreation, Auckland University of Technology, Auckland, New Zealand

**Keywords:** Accelerometer, Activity classification, Human activity recognition, Machine learning, Prediction, Validation

## Abstract

**Background:**

Accurately measuring energy expenditure during physical activity outside of the laboratory is challenging, especially on a large scale. Thigh-worn accelerometers have gained popularity due to the possibility to accurately detect physical activity types. The use of machine learning techniques for activity classification and energy expenditure prediction may improve accuracy over current methods. Here, we developed a novel composite energy expenditure estimation model by combining an activity classification model with a stride specific energy expenditure model for walking, running, and cycling.

**Methods:**

We first trained a supervised deep learning activity classification model using pooled data from available adult accelerometer datasets. The composite energy expenditure model was then developed and validated using additional data based on a sample of 69 healthy adult participants (49% female; age = 25.2 ± 5.8 years) who completed a standardised activity protocol with indirect calorimetry as the reference measure.

**Results:**

The activity classification model showed an overall accuracy of 99.7% across all five activity types during validation. The composite model for estimating energy expenditure achieved a mean absolute percentage error of 10.9%. For running, walking, and cycling, the composite model achieved a mean absolute percentage error of 6.6%, 7.9% and 16.1%, respectively.

**Conclusions:**

The integration of thigh-worn accelerometers with machine learning models provides a highly accurate method for classifying physical activity types and estimating energy expenditure. Our novel composite model approach improves the accuracy of energy expenditure measurements and supports better monitoring and assessment methods in non-laboratory settings.

**Supplementary Information:**

The online version contains supplementary material available at 10.1186/s12966-024-01646-y.

## Background

An individual’s physical behaviour includes physical activity and sedentary behaviour. Both are known to be important and modifiable behavioural factors affecting human health [1, 2]. The underlying dimensions of physical behaviour include intensity, duration, domain and type of activity or posture [3]. An important component of physical activity intensity is energy expenditure. Activity-related energy expenditure is the most variable component of total energy expenditure and can be modified by changes in physical behaviour [4]. Changes in energy expenditure can have a significant impact on energy balance and contribute to successful weight management [5]. Recent evidence from the UK Biobank suggests a strong association between activity-related energy expenditure and the incidence of type 2 diabetes [6]. In addition, accurate measures of energy expenditure can be incorporated into closed-loop insulin delivery systems and continuous glucose monitoring systems to improve glycaemic control in people with diabetes [7]. Thus, more accurate measurement of energy expenditure can help to understand energy balance and develop effective interventions to prevent and manage highly prevalent diseases such as obesity or type 2 diabetes.

While energy expenditure can be accurately estimated in the laboratory using various methods such as indirect calorimetry, it remains challenging in free living conditions [4]. The Doubly Labelled Water method is considered the gold standard for assessing free-living energy expenditure, but it remains costly, requires advanced infrastructure, and therefore cannot be easily scaled for large-scale use in research and medical settings.

Wearable devices such as accelerometers and smartwatches are now widely used to assess physical activity and can potentially be used to estimate free-living energy expenditure based on acceleration parameters or heart rate. However, existing wearable systems and estimation methods have shown limited accuracy in estimating energy expenditure [8–10]. Recent advances in the accuracy of accelerometer-based energy expenditure estimation have been made by using machine learning-based algorithms to classify activity types and combining these with activity-specific estimation models [11, 12]. Previous research also suggests that stride-segmented estimation methods based on lower body kinematics can be used for more accurate estimation of energy expenditure [13]. Most of these novel approaches have been developed and applied to hip- and wrist-worn accelerometers or require the use of multiple sensor placements.

Thigh-worn accelerometers are increasingly being used in large epidemiological studies due to the ability to accurately classify a range of activity types and postures using data-driven algorithms and raw acceleration signals [3, 14, 15]. A variety of machine learning techniques have been applied to thigh-worn accelerometer data, mainly focusing on activity type and posture classification [16–19] and intensity categories [20]. To date, a small number of studies have attempted to estimate energy expenditure from thigh-worn accelerometry, primarily using simple regression techniques on aggregated acceleration metrics or cadence [10, 21–23].

The recent success of more advanced energy expenditure estimation approaches, coupled with the ability to accurately classify activity type and available information on thigh kinematics, suggests the potential for more accurate energy expenditure estimation using the raw acceleration signal from a thigh-worn sensor. The aim of this study was to train a composite machine learning model to estimate energy expenditure from raw thigh accelerometer data by applying activity classification and stride segmentation to the raw accelerometer data, and to validate the model’s performance against indirect calorimetry.

## Methods

**Fig. 1:**
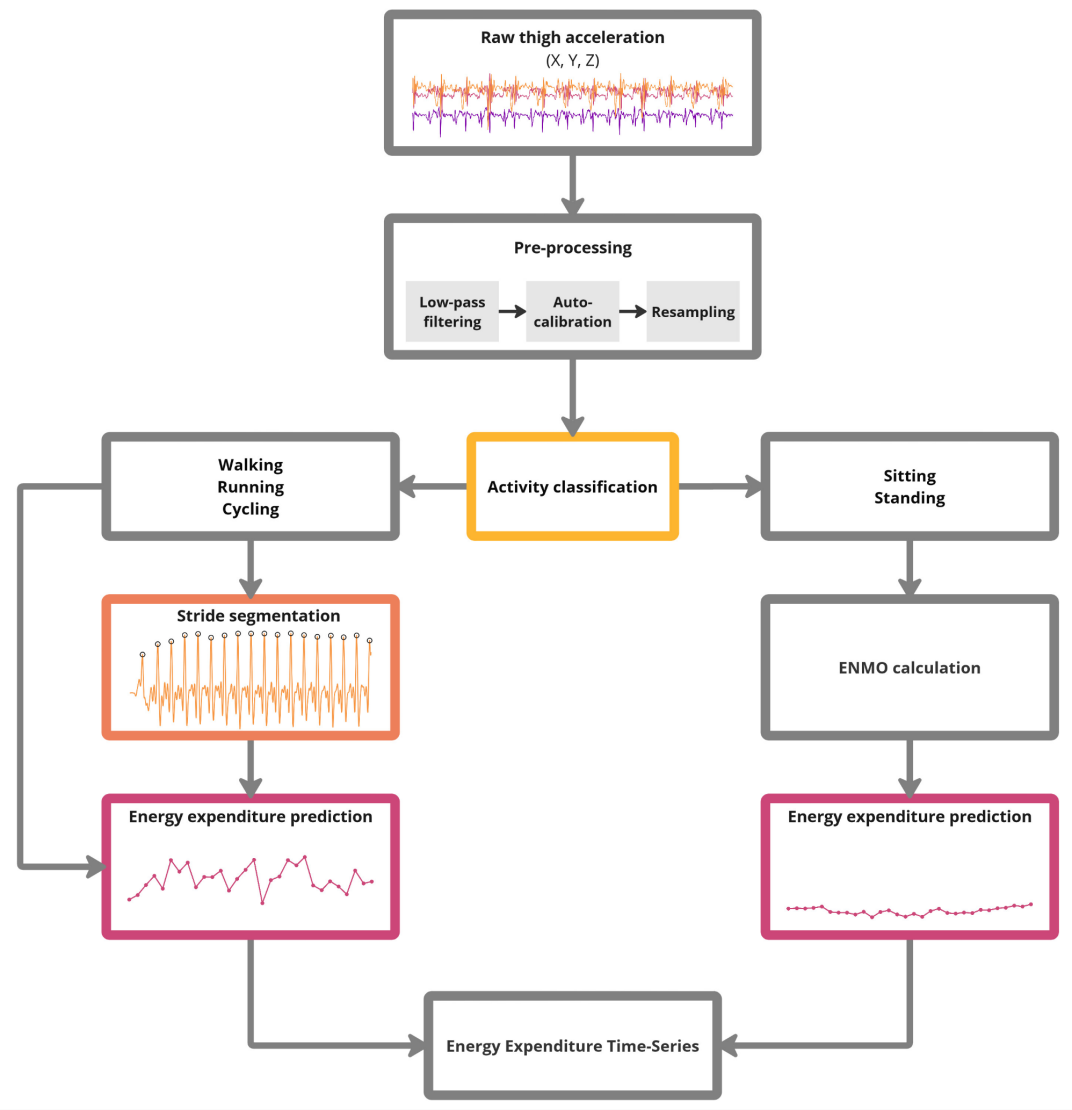
Processing steps comprised in the CATSE3 model. Raw triaxial acceleration data is required as input and a time-series with energy expenditure predictions is the resulting output

We developed a composite activity type and stride-specific energy expenditure estimation (CATSE3) model to enable energy expenditure estimation based on raw 3D acceleration. The multi-model approach involves several key steps and algorithms which are outlined in Fig. 1:

1. **Pre-processing**: The raw acceleration signal is resampled, auto-calibrated and low-pass filtered using an eighth-order Butterworth filter.
2. **Activity classification**: A classification algorithm identifies the activity type based on 4-second epochs of time-series 3D acceleration data.
3. **Stride segmentation**: Individual strides are identified within sequences of walking, running, and cycling. The 3D acceleration data is segmented into individual strides.
4. **Energy expenditure estimation**: Depending on the predicted activity type, energy expenditure is estimated based on individual strides (for walking, running, and cycling) or aggregated acceleration metrics (for sitting and standing).

### Sample population

The study included data collected from 69 healthy adults divided into two independent samples. Data from the training sample (*n* = 49; 45% female; age = 26.0 ± 6.3 years; BMI = 23.0 ± 2.6 kg/m²) were used to develop the processing pipeline and train the energy expenditure models. Data from the test sample (*n* = 20; 60% female; age = 23.2 ± 3.8 years; BMI = 23.4 ± 1.8 kg/m²) were used only to evaluate the developed approach.

Participants were recruited via newsletters, social media, and posters at the university campus. Participants had no known disability and were free to engage in physical activity. Each participant provided written informed consent prior to participation in the study, which was approved by the Ethics Committee of the German Sport University Cologne (ref. 142/2023). Participants were financially compensated for their time.

### Instrumentation

Triaxial acceleration was sampled at 100 Hz with a dynamic range of ± 8 g using an inertial measurement unit (AX6, Axivity Ltd, Newcastle upon Tyne, UK). Prior to each session, a calibration routine was performed in which the sensor was mounted inside a rectangular plastic cube. The cube was then placed on each side for 10 s to establish reference values for each axis. The device was attached directly to the skin on the lateral side of the participant’s dominant thigh, midway between the hip and knee. The device was first attached to a skin-friendly adhesive tape (Fixomull stretch; BSN medical GmbH, Hamburg, Germany) using double-sided adhesive tape, which was then placed on the skin. The device was secured with an elastic bandage (Peha-haft Color; Paul Hartmann AG, Heidenheim, Germany) wrapped around the thigh.

Walking and running were performed on an instrumented treadmill (quasar med; h/p/cosmos sports & medical GmbH, Nussdorf-Traunstein, Germany) and cycling was performed on a stationary bicycle ergometer (ergoselect 100; ergoline GmbH, Bitz, Germany). Indirect calorimetry was used to estimate reference energy expenditure via gas exchange using a metabolic cart (Metalyzer 3B, Cortex Biophysik GmbH, Leipzig, Germany). The metabolic cart was calibrated prior to each session following manufacturer instructions.

### Activity protocol

Each subject was asked to complete a standardised activity protocol including sitting, standing, walking, running, and cycling. The protocol varied slightly between the training and validation samples to introduce additional variability (Table 1). It was designed to include a range of basic activities and intensities while adhering to best practice recommendations [24]. Participants were allowed to skip any activity condition that they were unable to complete. All treadmill walking and running conditions were performed at a 1% incline to account for reduced energy expenditure, unless otherwise stated [24]. For all cycling conditions, revolutions per minute (rpm) were chosen ad libitum between 60 and 80 rpm. Each activity condition lasted 6 min. For the walking, running, and cycling conditions, participants had a 5-min rest period between each condition.

**Table 1:** Activity protocol for the training and validation group

	Training	Validation
Activity	Intensity / Speed	
Sitting		Freely
Standing	Freely	Freely
Walking (km/h)	2.1	2.5
	2.9	3.3
	3.7	4.1
	4.5	
	3.7 with 6% incline	3.3 with 6% incline
Running (km/h)	7.5	8
	8.5	9
	9.5	10
	10.5	
	9.5 with 6% incline	8 with 6% incline
Cycling (W)	30	50
	60	75
	90	100
	120	
	150	

### Data processing

Oxygen uptake and carbon dioxide output were measured on a breath-by-breath basis throughout the study and converted to energy expenditure using Weir’s equation [25]. Steady state energy expenditure was calculated by averaging all breaths over the last three minutes of each condition and expressed as kilocalories per minute per kilogram of body weight.

Raw acceleration data were downloaded from the sensors using OMGUI v1.0.0.30 [26] and saved as comma-separated values (CSV) files. The raw data were pre-processed using the actipy package v3.0.5 [27]. First, the acceleration data were low-pass filtered using an 8th order Butterworth filter with a cut-off frequency of 20 Hz. The data were then auto-calibrated against local gravity using a validated approach [28] and resampled to ensure a sampling rate of 100 Hz. The acceleration data were then partitioned into non-overlapping 4-second intervals.

### Classification of activity types

We trained a deep learning-based activity classification model to predict the activity type based on 4-second intervals of 3D acceleration data. To train the classification model, we used pooled data from three existing accelerometer datasets, totalling *n* = 69 healthy adult participants, independent of the data collected in this study. The characteristics of each dataset and the training process are detailed in Additional File 1. The pooled dataset included various laboratory and free-living physical activities, including walking, running, standing, sitting, lying down, and cycling. Reference labels for the activity type were obtained either from direct observation or from video annotation.

A supervised machine learning approach was used, where the model was trained on labelled data (i.e. 3D acceleration data and corresponding activity type label). During the training process, the model parameters are iteratively adjusted to minimise a given loss function corresponding to the overall classification error. We chose a hybrid Convolutional Neural Network (CNN) and Bidirectional Long-Short Term Memory (BiLSTM) model architecture to predict the probability of each activity class for a given 4-second interval. The hybrid architecture allows the deep learning model to extract spatial features based on the CNN layers while also learning temporal patterns using the BiLSTM layer. Previous research has successfully applied a hybrid CNN-BiLSTM approach to classify sedentary behaviour from hip-worn accelerometer data [29]. Model training and optimisation was performed using Keras [30] and KerasTuner [31]. We chose a window size of 4 s based on previous findings [16] and the time required to capture at least one full stride during slow walking.

### Segmentation of strides

For each continuous sequence (i.e. one or more 4-second segments) of walking, running, and cycling, the individual strides were identified and segmented using an activity-specific approach. First, the acceleration signal is low-pass filtered using a fourth order Butterworth filter. For running and cycling sequences, the acceleration data collected along the x-axis (i.e. the vertical axis of the thigh when standing still) is low-pass filtered with a cut-off frequency of 5 Hz. Similarly, for walking, the acceleration along the z-axis (sagittal axis) is low-pass filtered with a cut-off frequency of 5 Hz. After filtering, each of these signals is passed to a peak detection algorithm which identifies strides as peaks in the respective acceleration signal. In a final step, the acceleration data of each stride is discretised into an equal number of samples by splitting it into 30 bins using Fast Fourier Transform [13].

### Estimation of energy expenditure

A hybrid temporal convolutional network (TCN) deep learning model was trained to predict energy expenditure during running, walking, and cycling using the 3D acceleration of a stride and activity type as model inputs. The TCN model architecture [32] allows learning from sequential data and has previously been used to successfully estimate gait events based on raw time-series data from a single inertial measurement unit (IMU) worn on the shank [33]. We used the KerasTCN implementation [34] for model training. The model architecture and training procedures are described in more detail in Additional File 2.

In addition, we developed a number of different linear regression models to estimate energy expenditure based on the Euclidean Norm Minus One (ENMO) and the Mean Amplitude Deviation (MAD), which were calculated over each 4-second epoch. For baseline comparison, two simple linear regression models were fitted to the training set data to predict energy expenditure as the dependent variable using either ENMO or MAD. Two additional models were fitted by further including the activity type and its interaction effect with the acceleration metric (i.e. ENMO or MAD). These activity-specific linear regression models allowed the use of aggregated acceleration metrics to be compared with the use of more detailed stride-specific acceleration signals in the hybrid TCN model. The CATSE3 approach integrates the hybrid TCN model for walking, running, and cycling sequences and the ENMO-based activity-specific regression model for sitting and standing sequences.

### Statistical analysis

The classification performance for the trained CNN-BiLSTM activity classification model as well as the accuracy of the overall energy expenditure model was evaluated on the test dataset to provide robust estimates based on unseen data.

For the classification, the metrics used for evaluation included overall accuracy, recall, precision, and F_1_ score. The overall accuracy was calculated as the number of true positive predictions divided by the total number of samples. Recall was calculated as the number of true positives divided by the sum of true positives and false negatives. Precision was calculated as the number of true positives divided by the sum of true and false positives. The F_1_ score was calculated as the harmonic mean of recall and precision.

The metrics used to evaluate the accuracy of the energy expenditure estimation include the root mean squared error (RMSE), the mean absolute percentage error (MAPE), the coefficient of determination (R²), bias and 95% limits of agreement.

All data processing, model training and analysis were performed using Python v.3.11 [35]. The code used for analysis and the resulting models are available in the public Zenodo repository (10.5281/zenodo.13477127).

## Results

### Activity classification

**Fig. 2:**
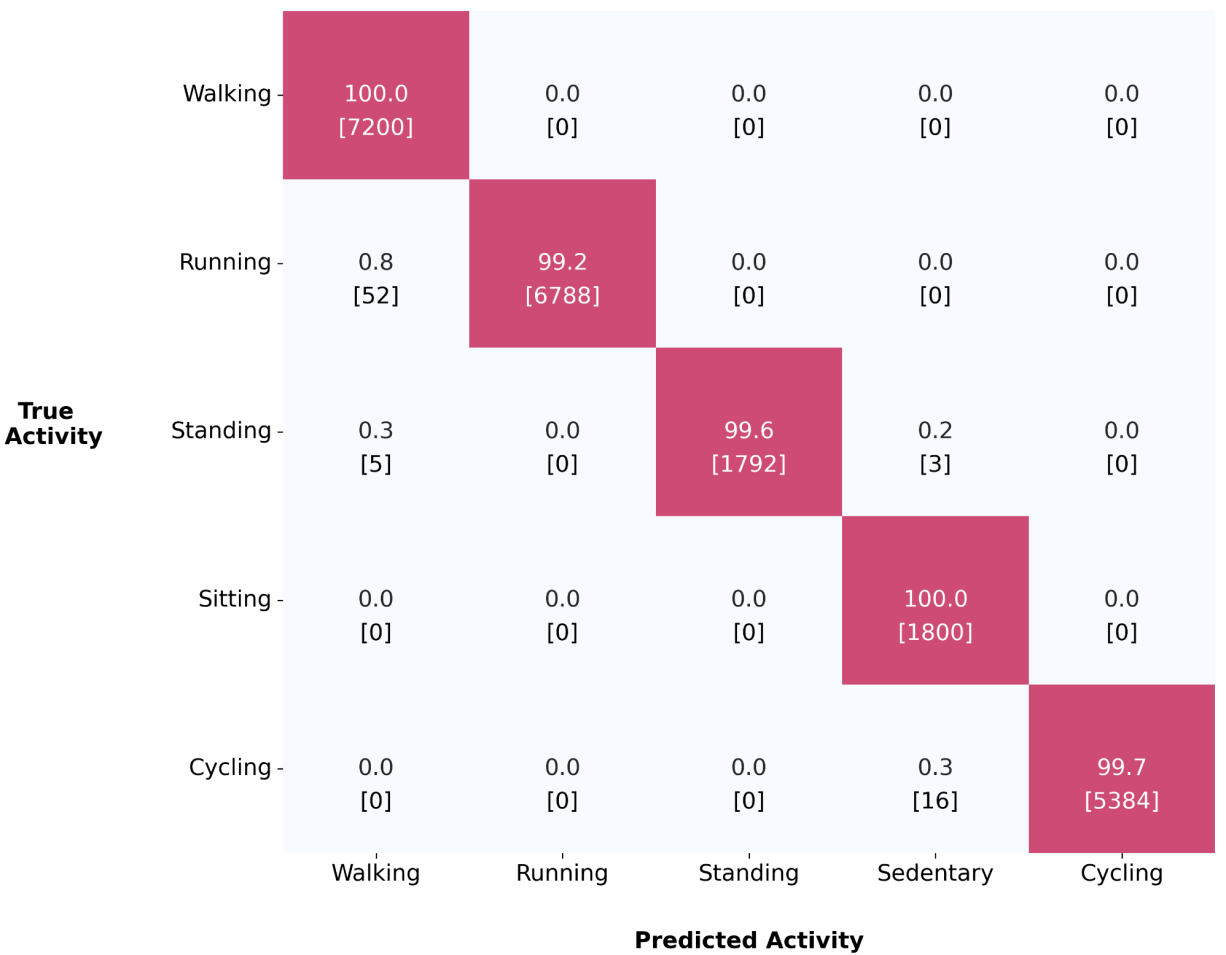
Confusion matrix with the ground truth activity classes and classes predicted by the CNN-BiLSTM classification model for the test set (*n* = 20 participants; 23,040 samples). Values presented are row percentages with the number of samples in brackets

The activity classification model achieved an overall accuracy of 99.7% with F_1_ scores > 0.99 across all five activity types when applied on the test set (*n* = 23,040 activity samples). Sensitivity ranges from 0.99 for running to 1.00 for walking and sitting (Fig. 2).

### Energy expenditure estimation

**Fig. 3:**
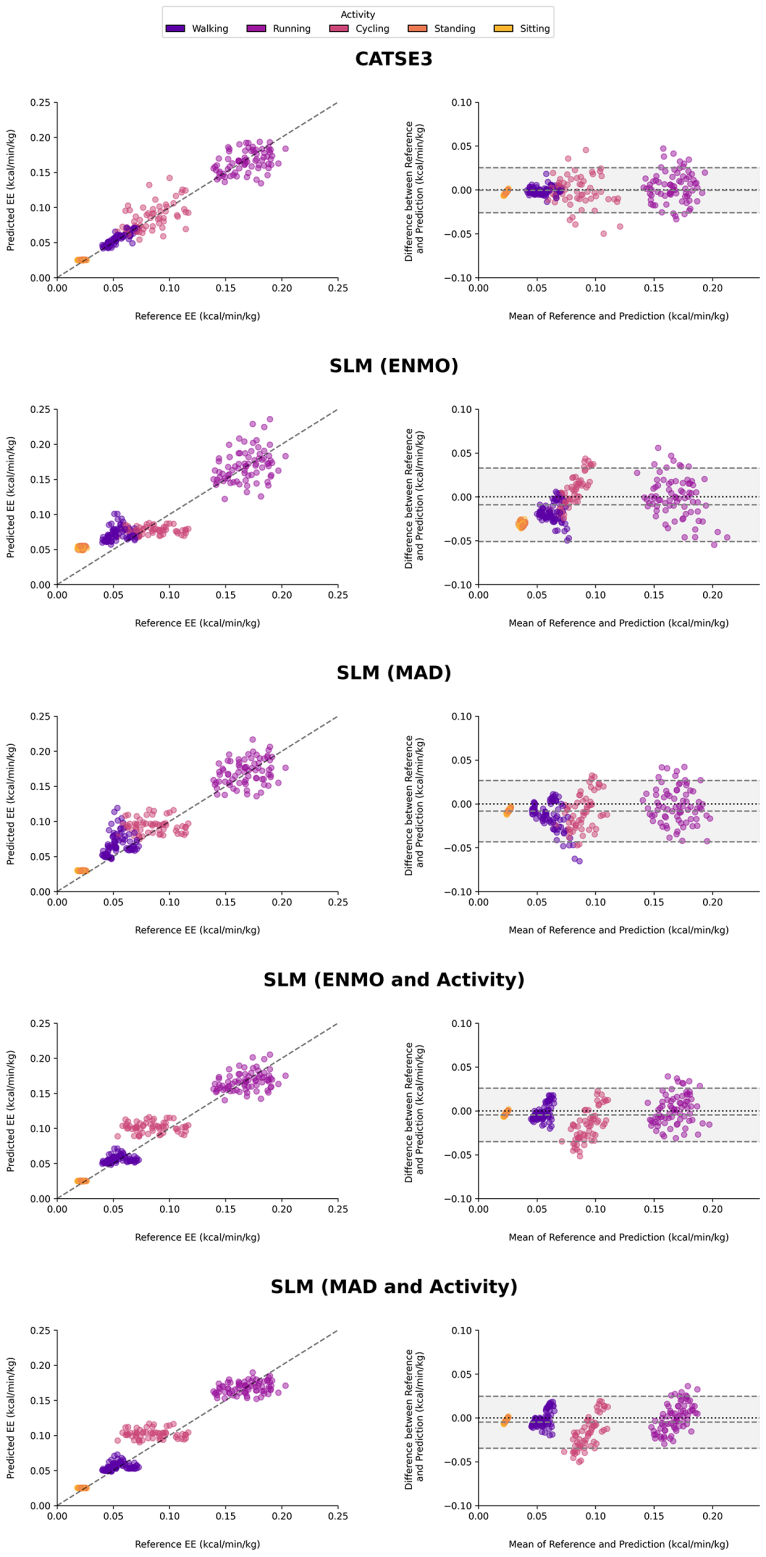
Scatterplots showing the relationship between the model energy expenditure predictions and the reference energy expenditure

The simple linear regression model using the ENMO metric showed the highest estimation error with a RMSE of 0.023 kcal/kg/min and MAPE of 41.5%. The stride and activity type-based algorithm (CATSE3) achieved the lowest RMSE of 0.013 kcal/kg/min and MAPE of 10.9% (Table 2). The R² values ranged from 0.828 for the simple ENMO model to 0.945 for the CATSE3 algorithm. The prediction of the CATSE3 model showed a negative bias close to zero (< 0.001 kcal/kg/min), whereas the predictions of all other models showed a negative bias of ≥ 0.005 kcal/kg/min (Fig. 3).

Across all walking conditions, the CATSE3 model achieved a MAPE of 6.6%, while the regression models including the activity type achieved MAPEs of 15.0% (ENMO + activity type) and 14.5% (MAD + activity type). MAPE values for running ranged from 7.9% (CATSE3 and MAD + activity type) to 10.5% (ENMO), while for cycling they ranged from 16.1% (CATSE3) to 29.6% (MAD + activity type). Post-hoc Pearson correlation analysis showed negative correlations between the participants height (*r* = -0.69) and weight (*r* = -0.47) with the prediction error in cycling.

**Table 2:** Error metrics for the energy expenditure estimation models. RMSE = root mean squared error; MAPE = mean absolute percentage error; R² = coefficient of determination; ENMO = euclidean norm Minus one; MAD = Mean Amplitude deviation

Model	Bias	95% Limits of Agreement		RMSE (kcal/kg/min)	MAPE (%)	*R*²
		Lower limit	Upper limit			
CATSE3	-0.001	-0.026	0.025	0.013	10.9	0.945
ENMO	-0.009	-0.051	0.033	0.023	41.5	0.828
MAD	-0.008	-0.043	0.027	0.020	24.2	0.876
ENMO + activity type	-0.005	-0.035	0.026	0.016	16.7	0.916
MAD + activity type	-0.005	-0.035	0.025	0.016	16.4	0.919

## Discussion

**Fig. 4:**
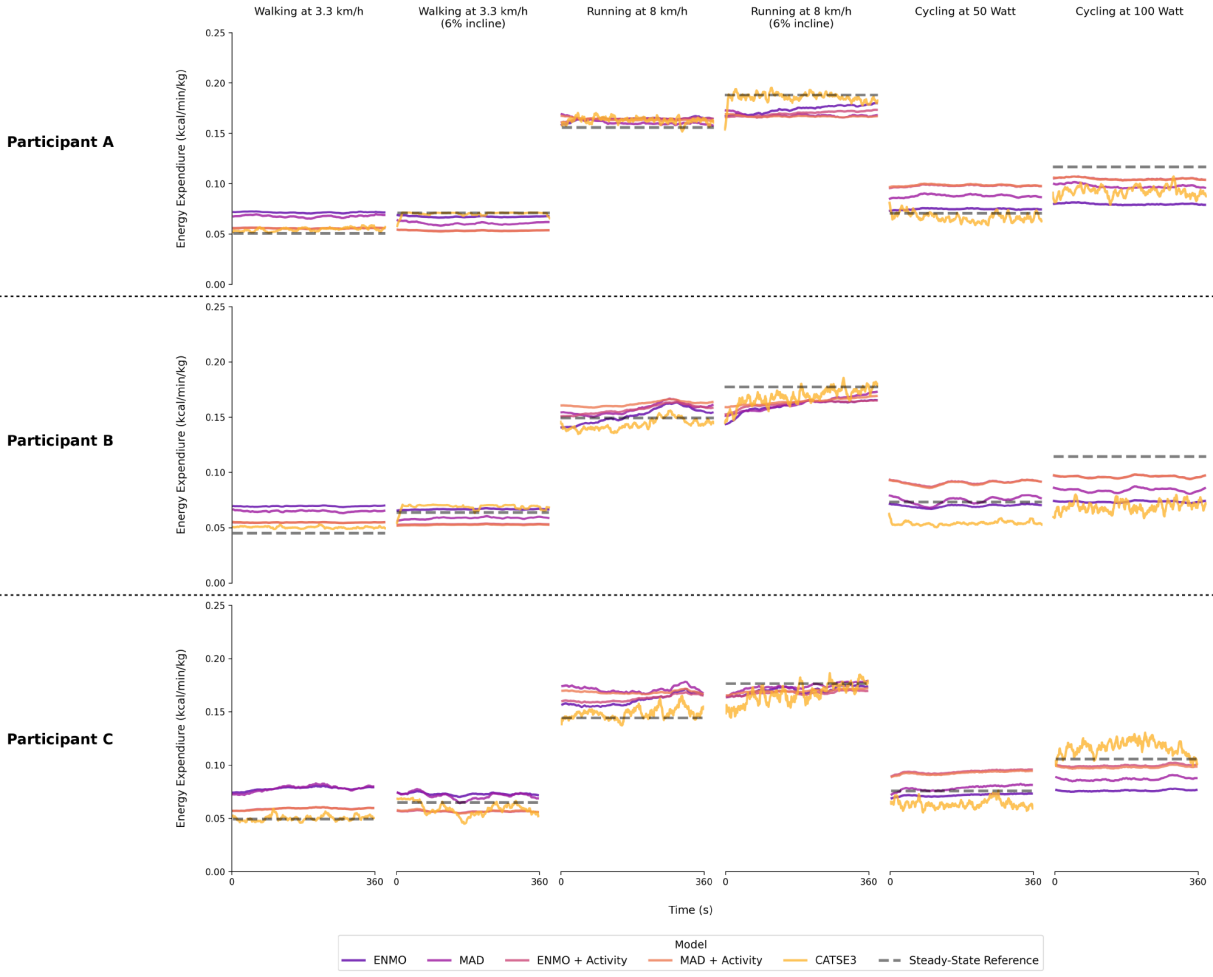
Predicted energy expenditure for different participants as well as walking, running, and cycling conditions

Our study aimed to develop and test a new approach for estimating energy expenditure based on raw 3D acceleration data from wearable accelerometers attached to the thigh. We successfully trained a deep learning algorithm on a pooled accelerometer dataset to classify basic activity types based on raw 3D acceleration data. By combining the activity classification algorithm with a stride-specific energy expenditure estimation approach, the CATSE3 model achieved a MAPE of 10.9% across different activities and intensities. In comparison, the activity-specific linear regression models showed a higher relative error (≥ 16.4%) and the simple linear regression models without the activity type showed more than twice the relative error (≥ 24.2%). These findings are consistent with results from previous research using a stride-specific model based on input data from two IMUs worn on the thigh and the shank [13]. The information contained in the raw 3D acceleration data of each stride cycle allows the CATSE3 model to distinguish between flat and inclined walking and running. This is probably due to small changes in the 3D acceleration curve during inclined gait. In comparison, the models based on the time-aggregated ENMO and MAD acceleration metrics do not appear to capture the increased energy expenditure associated with inclined walking and running (Fig. 4).

A more recent model based on wrist-worn IMU data combined an activity classification algorithm with walking speed estimation to predict energy expenditure during walking, sitting, and standing with an overall MAPE of 15% [11]. Previous research proposed a simple modelling equation based on the triaxial vector magnitude of a thigh-worn sensor to estimate energy expenditure during walking [22]. However, the model showed a MAPE of 18% over a range of walking speeds, whereas the CATSE3 model in our study showed a substantially lower MAPE of 6.6%. Importantly, the estimates of energy expenditure during cycling (MAPE = 16.1%) were not as accurate as during walking and running (7.9%). It appears that the model does not capture variations in energy expenditure during cycling very well (Fig. 3), which is consistent with previous research using hip-worn accelerometer data [12].

### Strengths and limitations

The activity protocol for this study followed recent guidelines for wearable energy expenditure validation studies [24]. The combination of multiple activity types, intensities, and flat and inclined treadmill activities allowed all models to be trained and evaluated over a wide range of activity conditions. However, additional activities such as stair climbing, or downhill walking and running were not included in this study.

It is important to recognise that the CATSE3 model and the mapping of energy expenditure to the stride acceleration time series are based on data from treadmill walking and running as well as stationary cycling. However, there may be differences in the stride acceleration profiles of the thigh in free-living conditions, that may affect the transferability of our results. Previous research suggests that the biomechanics of walking and running on a treadmill are largely comparable to those on the ground, but there are minor differences in spatiotemporal (i.e. shorter stride length and higher cadence) and sagittal plane kinematics (increased hip and knee flexion angles) [36–38]. The impact of these differences on thigh acceleration curves is currently unknown, but warrants caution and further investigation.

Our study benefited from the integration of different datasets for model training and evaluation. We trained our activity classification model on an independent dataset that included data from several existing studies in different contexts, including laboratory and free-living activities. Therefore, we expect the classification model to be reasonably robust, as evidenced by the high overall classification accuracy both during model development (99.3% for the hold-out set) and on the test dataset (99.7%). However, the classification performance may not be indicative of actual performance in real-world conditions and needs to be further evaluated, ideally using an independent dataset with free-living activities and a more heterogeneous sample. In general, our sample for this study is considered homogeneous in terms of BMI, age, and health status. Therefore, the generalisability of our results to other populations, such as children, older adults, or people with gait disorders, may not be given.

### Future investigations

It is currently unknown how the model performs for undefined activities and intensities outside the range included in the study (e.g. very fast walking). The inclusion of additional activity types (e.g. climbing stairs, lying in bed) and a wider range of walking and running speeds is likely to improve estimation accuracy. Future iterations of the model should therefore consider extending the range of activity types and account for unknown activity types.

Another way to improve the accuracy of the model may be to include body weight and height as additional input variables, as both are related to energy expenditure and the magnitude of the prediction error [22]. In addition, the estimation of energy expenditure during sitting and standing may be improved by integrating an estimate of the basal metabolic rate rather than including only an aggregated acceleration metric [13].

## Conclusions

To our knowledge, the CATSE3 model is the first energy expenditure model that uses both activity classification and stride segmentation to estimate energy expenditure based solely on thigh acceleration data. With this study, we provide the physical activity research community with several open-source energy expenditure estimation algorithms for thigh-worn accelerometers of varying complexity.

Our results show that stride specific information contained in the 3D acceleration signal can be used in combination with activity type information to achieve a lower estimation error when compared to simpler estimation approaches currently used in physical activity research. Energy expenditure estimates based on activity and stride-specific information appear to be more accurate than approaches using aggregated acceleration metrics such as ENMO or MAD. The integration of available and validated activity classification algorithms should be encouraged in future research on energy expenditure estimation methods using thigh-worn accelerometry.

## Electronic supplementary material

Below is the link to the electronic supplementary material.

Supplementary Material 1: Additional file 1 (.pdf)- Training of the activity classification model.

Supplementary Material 2: Additional file 2 (.pdf)- Training of the stride-specific energy expenditure model.

## Data Availability

The code and anonymised data necessary to reproduce the results of this study are available from the Zenodo repository (10.5281/zenodo.13477127). The data includes raw data and custom Python code necessary to reproduce the results, tables, and figures of this study. The data used to train the activity classification algorithm can be requested from the respective authors.

## Abbreviations

CATSE3: Composite Activity Type and Stride-specific Energy Expenditure Estimation
CNN-BiLSTM: Convolutional Neural Network - Bidirectional Long-Short Term Memory
ENMO: Euclidean Norm Minus One
MAD: Mean Amplitude Deviation
MAPE: Mean Absolute Percentage Error
RMSE: Root Mean Squared Error
TCN: Temporal Convolutional Network
