# Supplementary material for: Composite activity type and stride-specific energy expenditure estimation model for thigh-worn accelerometry
Source: Int J Behav Nutr Phys Act. 2024 Sep 10;21:99. doi: 10.1186/s12966-024-01646-y (PMC11389320; doi:10.1186/s12966-024-01646-y)
Supplement: Supplementary file 1 — Supplementary Material 1: Additional file 1 (.pdf)- Training of the activity classification model. [file 12966_2024_1646_MOESM1_ESM.pdf]

Training of the activity classification model

The individual datasets used to train the CNN-BiLSTM classification model are described in Table S1. All underlying studies have been approved by the respective ethics committee (Dataset A: German Sport University Ethics Committee [ref. 107/2023]; Datasets B and C: AUT University Ethics Committee [ref. 17/220 and 18/99]). For each study, participants gave written informed consent prior to data collection.

Table S1 Characteristics of the individual datasets used for training the CNN-BiLSTM activity classification algorithm.

Dataset	A	B	C
Data source	Lendt et al. 2024 (unpublished)	Narayanan et al. 2020	Stewart et al. 2018
Sample size	21	15	33
Participant characteristics	female = 42.9%<br>age = 26.7 ± 4.2 years<br>BMI = 23.4 ± 1.6 kg/m²	female = 66.7%<br>age = 31.5 ± 10.8 years<br>BMI = n/a	female = 51.5%<br>age = 42.4 ± 9.9 years<br>BMI = n/a
Setting	free-living and laboratory	free-living	laboratory
Activities	cycling, running, walking	cycling, lying, running, sitting, standing, walking	lying, reclining, running, sitting, standing, walking
Reference measure	direct observation	wearable video camera (annotated activity type)	stationary video camera (annotated activity type)
Sampling frequency and range	100 Hz; ± 8g	100 Hz; ± 8g	100 Hz; ± 8g
Accelerometer type	Axivity AX6	Axivity AX3	Axivity AX3

The distribution of activity types for each dataset is shown in Figure S1. Sitting and lying were grouped into a single ‘sedentary’ activity type. For each dataset, 4-second epochs with 75% overlap were created using a sliding window with a 1-second shift across each individual activity sequence. Only epochs with a single true label were considered, meaning that no ambiguous epochs containing two or more activities were created. Each of the final epochs included 400 data points of continuous 3D acceleration data and a single reference activity label (i.e., 100 Hz, four seconds).

Epochs from ~ 80% of the participants from each of the three datasets were selected for the training set and the remaining ~ 20% for the test set. A holdout sample from the training set (~ 20% of the participants in the training set) was used as a validation set for model optimisation. This resulted in a training set of 54,231 epochs from n = 41 participants across all three datasets. The validation and test set comprised 18,852 (n = 13) and 18,272 (n = 15) samples, respectively.

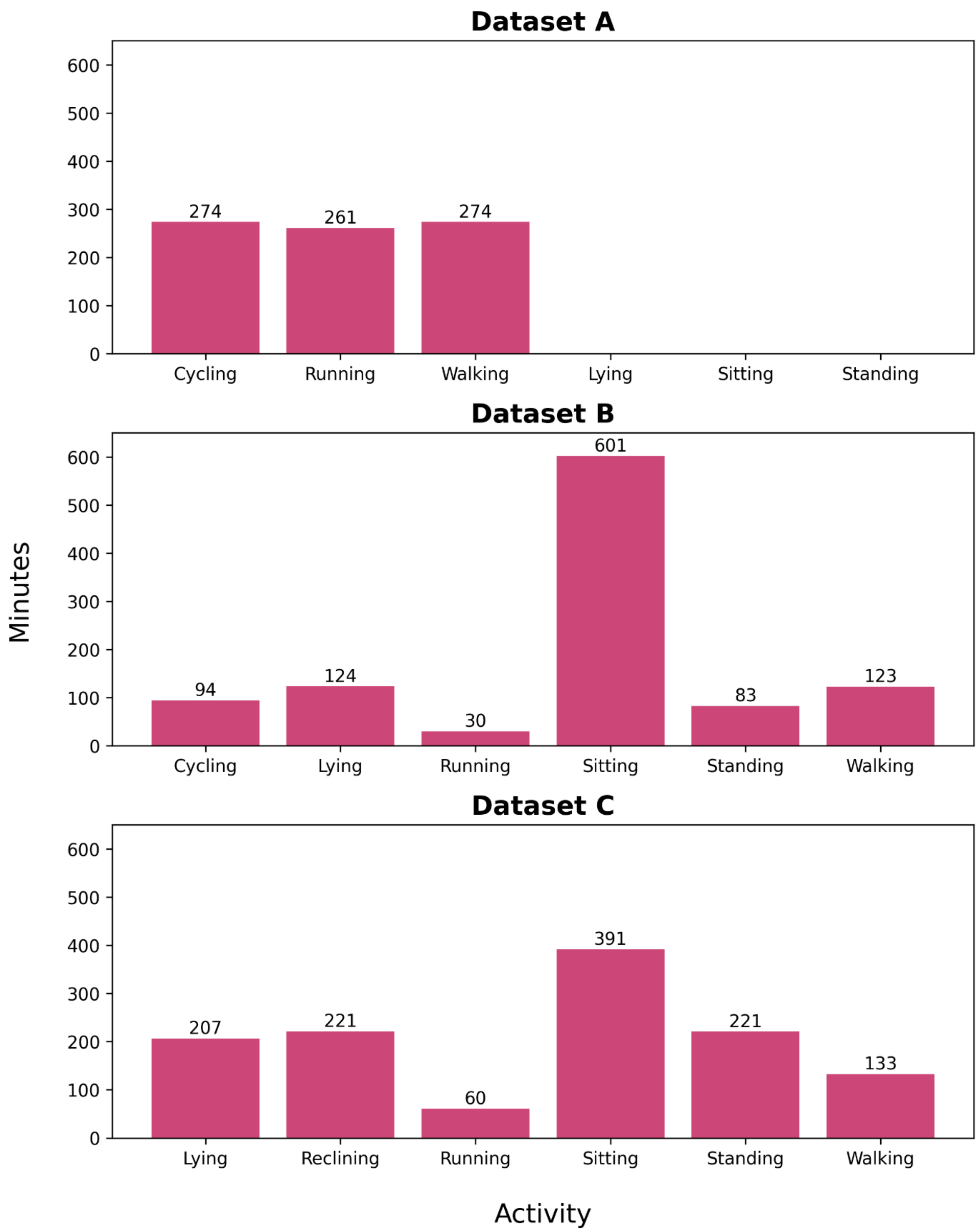

Figure S1 Time distribution for each activity label in Datasets A, B, and C.

First, we identified the best performing model architecture on the validation set using an iterative approach. Subsequently, the number of filters and kernel size of the

convolutional layers as well as the number of units in the BiLSTM layer were optimised during hyperparameter optimisation using KerasTuner [24] and a predefined search space (Table S2). Model training was performed using the class-weighted categorical crossentropy as the loss function and Adam optimisation with a learning rate of 1e-4.

Table S2 Hyperparameter search space used for the grid search to define optimal parameters for the CNN-BiLSTM activity classification model.

Hyperparameter	Values	Best set of values
Number of filters (first CNN layer)	32, 64	**64**
Kernel size (first CNN layer)	8, 16, 32	**32**
Number of units (BiLSTM layer)	16, 32, 64	**64**

The model architecture with the best performance (i.e. lowest loss) during the hyperparameter optimisation process was finally trained on the combined training and validation set, to evaluate its performance on the test set. The final trained model achieved an overall accuracy of 99.3% on the test set with F_1 scores ≥ 0.98 across all five activity classes. Figure S2 shows the confusion matrix for the model predictions on the test set.

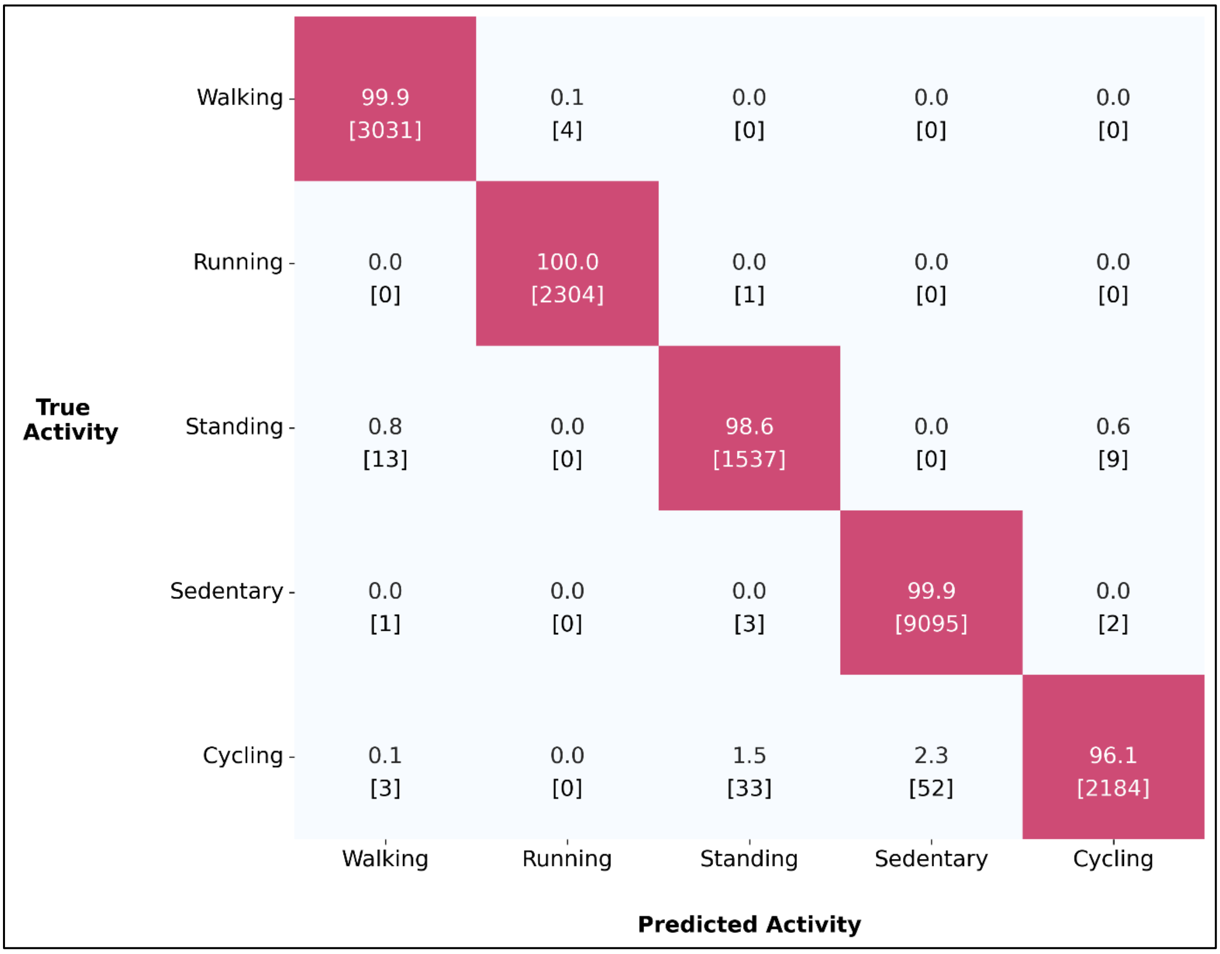

Figure S2 Confusion matrix with the ground truth activity classes and the classes predicted by the CNN-BiLSTM classification model for the test set (n = 15 participants; 18,272 samples). Values shown are row percentages with the number of samples in square brackets.
