# Supplementary material for: Composite activity type and stride-specific energy expenditure estimation model for thigh-worn accelerometry
Source: Int J Behav Nutr Phys Act. 2024 Sep 10;21:99. doi: 10.1186/s12966-024-01646-y (PMC11389320; doi:10.1186/s12966-024-01646-y)
Supplement: Supplementary file 2 — Supplementary Material 2: Additional file 2 (.pdf)- Training of the stride-specific energy expenditure model. [file 12966_2024_1646_MOESM2_ESM.pdf]

Training of the stride-specific energy expenditure model

For the purpose of training and optimising the model, the training sample data was initially split into a training set (n = 39; 208,825 strides) and a validation set (n = 10; 47,086 strides) using a random 4:1 split. The data included stride level accelerations for all walking, running, and cycling activities. Each sample (i.e. stride) contained 30 time points of 3D acceleration data (x, y, z) and the corresponding one-hot encoded activity class as features (93 features). The corresponding target values were the steady-state energy expenditure normalised by the participant's body weight.

The hybrid model architecture was based on a non-causal TCN layer including batch normalisation (Figure S3). The dilations for the TCN layer were set to [1, 2, 4, 8, 16].

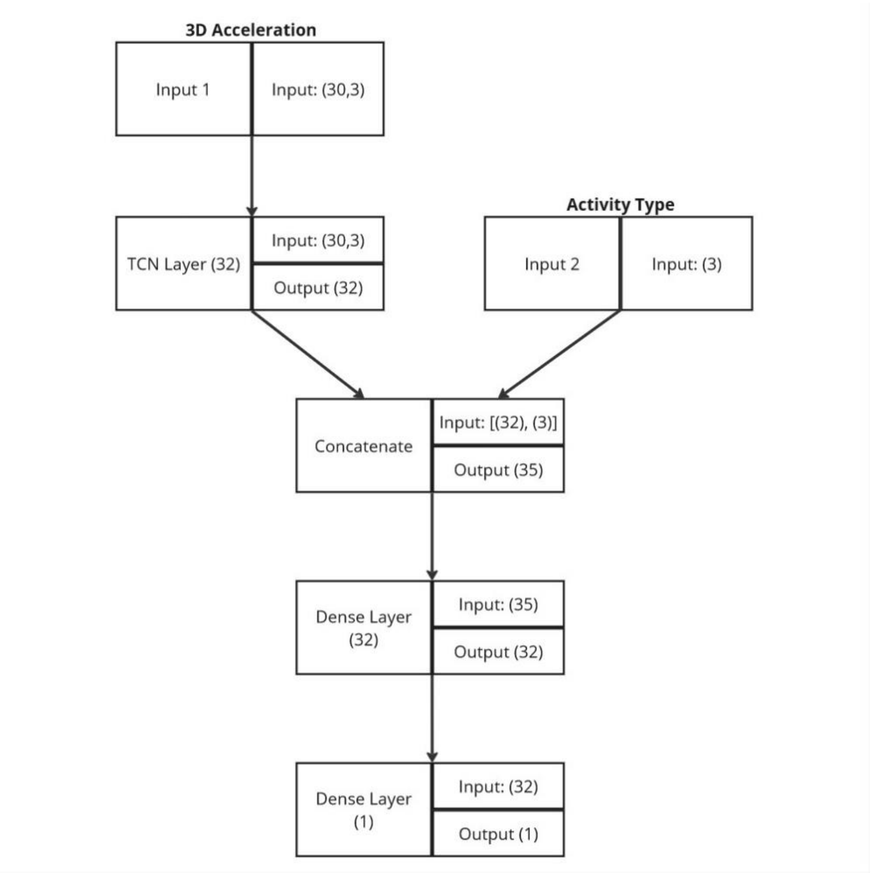

Figure S3 Hybrid TCN model architecture.

Hyperparameter optimisation was performed on the validation set to identify the optimal number of filters and kernel size for the TCN layer, as well as the number of units for the Dense layer, using a grid search with KerasTuner [23]. The model architecture with the best performing set of hyperparameters (Table S3) was then trained on the combined training and validation set. The mean absolute percentage error was used as the loss function and gradient descent-based optimization algorithm (Adam) was used to learn the model weights.

Table S3 Hyperparameter search space used for the grid search to define optimal parameters for the hybrid-TCN energy expenditure estimation model.

Hyperparameter	Values	Best set of values
Number of filters (TCN layer)	16, 32, 64	**32**
Kernel size (TCN layer)	2, 3, 4	**3**
Number of units (Dense layer)	16, 32, 64	**32**
